# Eiger triggers death from afar

**DOI:** 10.7554/eLife.01388

**Published:** 2013-09-24

**Authors:** Ginés Morata, Salvador C Herrera

**Affiliations:** 1 **Ginés Morata** is at the Centro de Biología Molecular Severo Ochoa, Universidad Autónoma de Madrid, Madrid, Spaingmorata@cbm.uam.es; 2 **Salvador C Herrera** is at the Centro de Biología Molecular Severo Ochoa, Universidad Autónoma de Madrid, Madrid, Spain

**Keywords:** apoptosis, TNF, signaling by apoptotic cells, JNK pathway, hair follicle cycle, cell death, *D. melanogaster*, Mouse

## Abstract

Cells undergoing programmed cell death release signals that can trigger the death of cells at remote locations.

**Related research article** Pérez-Garijo A, Fuchs Y, Steller H. 2013. Apoptotic cells can induce non-autonomous apoptosis through the TNF pathway. *eLife* **2**:e01004. doi: 10.7554/eLife.01004

**Image** Cell death in the *Drosophila* posterior wing disc compartment (red) induces cell death (blue) in the anterior compartment (green)

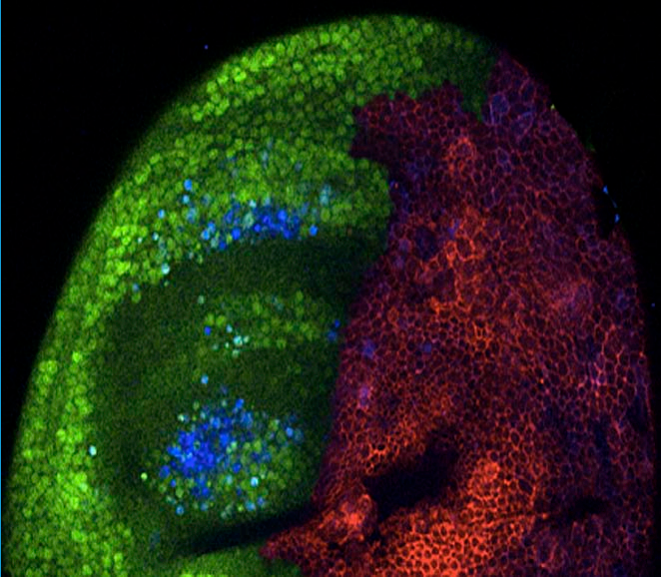

All animal cells contain the seeds of their own destruction: inactive forms of enzymes called caspases. Activation of these enzymes culminates in a process called programmed cell death. This process—which is also known as apoptosis—must be finely regulated as too much or too little apoptosis can disrupt development and has also been implicated in a number of human diseases.

The principal features of apoptosis are well understood (see a recent review by Fuchs and Steller, 2011). Traditionally, it has been regarded as a passive process in which the doomed cell is simply destroyed by the catalytic activity of the caspases. However, in recent years, it has become clear that dying cells interact with their healthy neighbours. In addition to sending the ‘find-me, eat-me signals’ that lead to their own elimination, they can also send signals that cause other cells to proliferate (Pérez-Garijo et al., 2004; Ryoo et al., 2004). Now, in *eLife*, Ainhoa Pérez-Garijo, Yaron Fuchs and Hermann Steller at the Rockefeller University reveal another remarkable property of apoptotic cells: namely that they can induce apoptosis from afar in cells that were not subjected to the initial apoptotic stimulus (Pérez-Garijo et al., 2013).

Pérez-Garijo, Fuchs and Steller performed the majority of their experiments in a *Drosophila* larval structure called the wing imaginal disc. This structure, which gives rise to the wings of the adult, is subdivided into two distinct lineage blocks, the anterior and the posterior compartment. The sophisticated methods of gene manipulation that are possible in *Drosophila* (Brand and Perrimon, 1993; Potter et al., 2010) allow an experimental analysis of apoptosis that cannot be performed in other organisms. Moreover, apoptotic cells can be kept alive indefinitely through the expression of a protein from baculoviruses called P35, which blocks the activity of the executioner caspases (Hay et al., 1994). These ‘undead’ cells have been used to reveal properties of apoptotic cells that are difficult to discern in normal apoptotic cells, which are short-lived (Pérez-Garijo et al., 2004; Ryoo et al., 2004). Another convenient feature of the wing disc is that no apoptosis occurs during normal development: this means that any apoptosis that is observed must be due to experimental intervention.

Pérez-Garijo et al. used the undead cell system to express the pro-apoptotic gene *hid* in the posterior compartment of the wing imaginal disc; as expected, it induced massive apoptosis in the posterior compartment, although the cells did not die. Unexpectedly, however, they also saw substantial apoptosis in the anterior compartment; that is, apoptosis propagated across the antero–posterior compartment boundary to cells in which the *hid* gene had not been activated. This phenomenon—which Pérez-Garijo et al. have named AiA, short for apoptosis-induced apoptosis—is not an artefact related to the use of undead cells, because the same phenomenon also occurred in cells not protected by P35. Moreover, apoptosis-induced apoptosis was seen in other imaginal discs but not, intriguingly, in the eye-antenna disc.

Now, consider the apoptotic program of *Drosophila*, outlined in Figure 1A. The induction of *hid* eventually leads to activation of the caspase Dronc and subsequently to activation of the effector caspase Drice. However, Dronc has an additional function: it also activates, by an unknown mechanism, a signalling cascade known as the JNK pathway: this upregulates the expression of *hid* (and other pro-apoptotic genes), establishing a positive feedback loop (Shlevkov and Morata, 2012).

**Figure 1.**
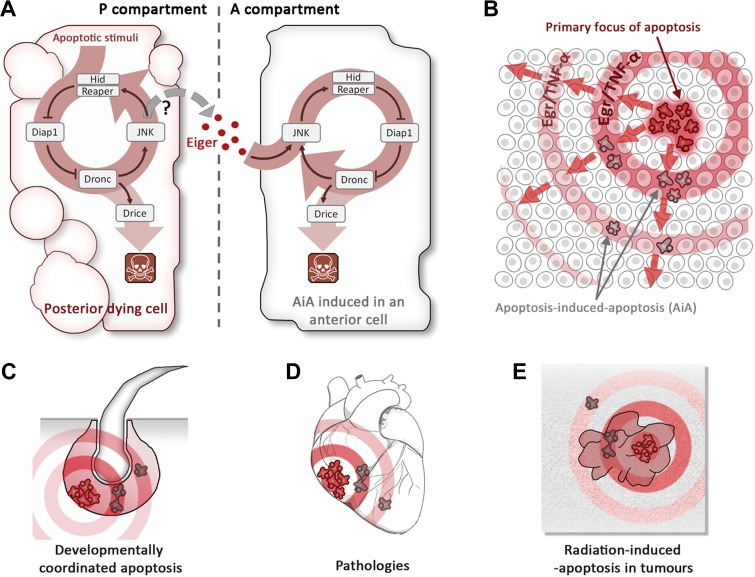
Apoptosis-induced apoptosis (AiA). (**A**) Introduction of the pro-apoptotic protein Hid into the posterior compartment (left) of the *Drosophila* wing imaginal disc triggers the propagation of apoptosis from the posterior to the anterior (right) compartment; introducing a protein called Reaper has a similar effect. The presence of Hid or Reaper in the posterior cells triggers the apoptotic loop: it degrades the anti-apoptotic protein Diap1, thus allowing activation of the caspase Dronc. This has two consequences: it activates the caspase Drice, which is responsible for actually killing the cell, and it upregulates the JNK pathway, which amplifies the activity of the genes that code for Hid and Reaper. The posterior cells produce a ligand called Eiger (Egr), which diffuses across the anterior–posterior border and activates the JNK pathway in anterior cells, triggering apoptosis. It is thought that the Eiger signal is produced by the JNK pathway itself, but the evidence is not conclusive (hence the question mark). (**B**) Schematic representation of AiA. The cells at the focal point of the initial apoptotic event emit death signals: Egr in flies or TNF-α in mammals. These signals diffuse away and induce secondary apoptosis in cells located at a distance from the primary focus. (**C**–**E**) are examples of scenarios in which AiA may play a role—these include normal processes such as the hair follicle cycle (**C**), pathological situations such as heart infarction (**D**), as well as radiation therapy in tumours (**E**).

The Rockefeller team found that the key to AiA is the upregulation of a protein called Eiger. This protein—which is similar to Tumour Necrosis Factor alpha (TNF-α) in mammals—is known to activate the JNK pathway (Moreno et al., 2002). Pérez-Garijo et al. went on to show that Eiger is produced in the posterior compartment and then travels to the anterior compartment, where it induces JNK signalling and subsequently activates the apoptotic loop (Figure 1A).

The involvement of the JNK pathway is of interest because this pathway is responsible for the proliferative signalling emanating from apoptotic cells (Pérez-Garijo et al., 2009). One could speculate that it is also implicated in the production of the Eiger signal, and hence apoptosis at a distance: the fact that increasing the level of JNK signalling in the posterior compartment also increases AiA in the anterior compartment supports this view (Figure 6 in Pérez-Garijo et al., 2013).

To explore whether apoptosis could propagate in other systems, Pérez-Garijo et al. examined the mouse hair follicle. The production of new hairs involves a cycle of growth, coordinated cell death (known as the catagen phase) and rest, and TNF-α signalling has been implicated in this cycle. Pérez-Garijo et al. confirmed that it is the apoptotic cells that produce TNF-α during the catagen phase, and that inhibiting TNF-α dramatically reduces apoptosis and disrupts hair follicle functioning. This is fully consistent with involvement of AiA during the mouse hair follicle cycle (Figure 1).

There are a number of situations, both normal and pathological, in which AiA may play a role. Processes such as the removal of the tadpole tail in amphibians or the interdigital webbing of many vertebrates are associated with collective apoptosis that may be coordinated by AiA. It may also underlie some human pathologies associated with massive apoptosis, such as cardiac infarction, cirrhosis or Alzheimer’s disease. In cancer therapy, AiA might have a role in the ‘bystander effect’’—the observation that cells that have not been exposed to radiation sometimes respond in the same way as nearby cells that have been exposed to radiation (Prise and O’Sullivan, 2009). It is possible that in these cases, the initial apoptotic stimulus triggers a death epidemic in which Eiger or TNF-α act as the propagating vectors.
